# EXPLORING THE SOCIO-SPIRITUAL MEANING AND COPING STRATEGIES OF OLDER ADULTS LIVING WITH CANCER IN NIGERIA

**DOI:** 10.1093/geroni/igad104.3337

**Published:** 2023-12-21

**Authors:** Obinna Odo, Kingsly Udeh, Jonathan Okolie, Runcie Chidebe

**Affiliations:** Scripps Centre, Miami University, Oxford, Ohio, United States; Scripps Centre, Miami University, Oxford, Ohio, United States; Scripps Centre, Miami University, Oxford, Ohio, United States; Scripps Centre, Miami University, Oxford, Ohio, United States

## Abstract

The growing cancer mortality, poor access to palliative care, treatment hesitancy, poor survivorship, and limited oncology healthcare infrastructure have been investigated in Sub-Saharan Africa. In Nigeria, the orthodox treatment and management of cancer is still greatly influenced by superstitious belief and spiritual connotations. Yet, the socio-spiritual and culturally sensitive meanings attached to cancer and treatment are still lacking in the literature. This study explored the socio-spiritual strategies of older adults living with cancer in Nigeria. The study adopted a qualitative approach and data were collected through a semi-structured interview with 15 older adults from the Oncology unit, University of Nigeria Teaching Hospital (UNTH) Enugu State, Nigeria. The data were managed and inductively coded using NVivo 12 and analyzed thematically. The findings of our study show that cancer is traditionally conceptualized as the aftermath of a disconnection with supernatural/ancestral forces. By implication, such misconceptions affect the level of dispositions and steps taken toward diagnosis and treatment. To this end, our study argues that blending spiritual needs, social support, and culturally sensitive cancer education could improve the quality of life and coping capacity of older adults living with cancer. The study recommends the infusion of socio-spiritual interventions to cancer treatment for older adults in Nigeria.

